# A Mechanistic Insight into the Pathogenic Role of Interleukin 17A in Systemic Autoimmune Diseases

**DOI:** 10.1155/2022/6600264

**Published:** 2022-05-17

**Authors:** Radjesh Bisoendial, Erik Lubberts

**Affiliations:** ^1^Department of Rheumatology and Clinical Immunology, Maasstad Hospital, Rotterdam, Netherlands; ^2^Department of Immunology, Erasmus Medical Center, Rotterdam, Netherlands; ^3^Department of Rheumatology, Erasmus Medical Center, Rotterdam, Netherlands

## Abstract

Interleukin 17A (IL-17A) has been put forward as a strong ally in our fight against invading pathogens across exposed epithelial surfaces by serving an antimicrobial immunosurveillance role in these tissues to protect the barrier integrity. Amongst other mechanisms that prevent tissue injury mediated by potential microbial threats and promote restoration of epithelial homeostasis, IL-17A attracts effector cells to the site of inflammation and support the host response by driving the development of ectopic lymphoid structures. Accumulating evidence now underscores an integral role of IL-17A in driving the pathophysiology and clinical manifestations in three potentially life-threatening autoimmune diseases, namely, systemic lupus erythematosus, Sjögren's syndrome, and systemic sclerosis. Available studies provide convincing evidence that the abundance of IL-17A in target tissues and its prime source, which is T helper 17 cells (Th17) and double negative T cells (DNT), is not an innocent bystander but in fact seems to be prerequisite for organ pathology. In this regard, IL-17A has been directly implicated in critical steps of autoimmunity. This review reports on the synergistic interactions of IL-17A with other critical determinants such as B cells, neutrophils, stromal cells, and the vasculature that promote the characteristic immunopathology of these autoimmune diseases. The summary of observations provided by this review may have empowering implications for IL-17A-based strategies to prevent clinical manifestations in a broad spectrum of autoimmune conditions.

## 1. Introduction

Interleukin 17A (IL-17A) represents a pleiotropic cytokine that has gained attention as signature cytokine of CD4^+^ T helper 17 (Th17) cells and has been put forward as critical determinant of psoriasis, a chronic relapsing T cell-mediated inflammatory disorder of the skin, and rheumatic musculoskeletal diseases like psoriatic arthritis and peripheral and axial spondylarthritis. In the last decade, a plethora of effective biological disease-modifying antirheumatic drugs (bDMARD) targeting the IL-23/IL-17A pathway has been developed, consisting of monoclonal antibodies against the common p40 subunit of IL-23/IL-12 and anti-IL-17A and the IL-23 p19 unit. Emerging data now indicates that IL-17A exert a wide range of functions that may be responsible for the development or exacerbation of systemic autoimmune diseases. Given the available data thus far, targeting IL-17A may be considered a novel strategy to prevent clinical manifestations in a broad spectrum of autoimmune conditions. In this review, we will focus on the pathophysiologic role of IL-17A in three major systemic autoimmune diseases including systemic lupus erythematosus (SLE), Sjögren syndrome (SS), and systemic sclerosis (SSC). For the sake of space restrictions, we will not address the role of IL-17A in other autoimmune diseases or rheumatic musculoskeletal diseases.

## 2. IL-17A: An Introduction to Its Immunological Functions

**Figure 1:**
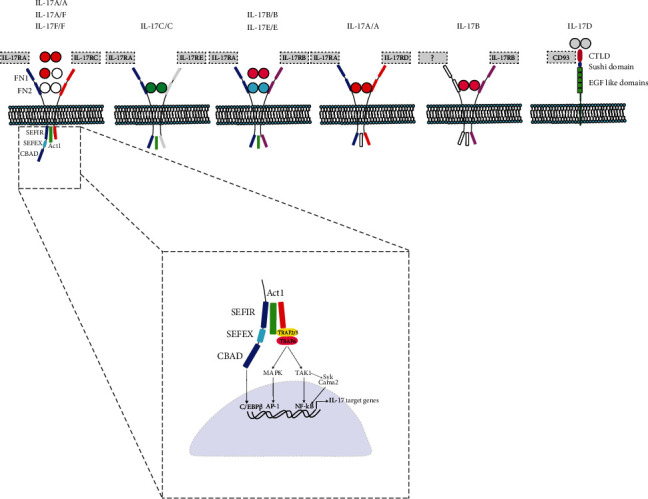
Schematic representation of IL-17 family of cytokines and receptors. IL-17A is the front runner in the IL-17 family of cytokines, comprising six homologues IL-17A to IL-17F in total. The IL-17 receptor family consists of five canonical IL-17 receptors, named IL-17RA to RE; CD93 has recently been identified as receptor for IL-17D. SEFIR: similar expression of fibroblast growth factor and IL-17Rs; SEFEX: SEFIR extension; CBAD: C/EBP*β* activation domain.

IL-17A is a front runner in the IL-17 family that comprises the six homologues IL-17A to IL-17F. IL-17A shares the greatest homology with IL-17F, where IL-17A and IL-17F represent the best studied members of the IL-17-family [1, 2]. Like other proinflammatory cytokines such as TNF, IL-17A displays AU rich repeats in the 3′untranslated region of its messenger RNA (mRNA). IL-17A has been originally identified as a T cell hybridoma-derived molecule CTLA-8 and shares a 57% amino acid sequence homology with a putative protein that was found in the T cell tropic *γ*-herpesvirus *saimiri* [3, 4]. Amongst the five canonical IL-17 receptors, named IL-17RA to RE, IL-17A signals as a homodimer IL-17A/A via the heterodimeric IL-17RA/RC receptor complex, which may also be engaged by the homodimer IL-17F/F (IL-17F) and the IL-17A/IL-17F heterodimer (IL-17A/F) [1] (see Figure 1). Of note, CD93 has recently been identified as receptor for IL-17D in group 3 innate lymphoid cells [5]. In terms of the hierarchy of activity, the most potent ligand of the IL-17RA/IL-17RC receptor complex is IL-17A/A, followed by IL-17A/F and IL-17F/F. Where the IL-17RA subunit is ubiquitously expressed, IL-17RC expression is restricted to nonhematopoietic epithelials and mesenchymal cell types [2]. Recently, IL-17RD has been suggested to represent an alternative receptor for IL-17A signaling, particularly in mouse and human keratinocytes [6]. The IL-17RA/RC receptor complex is expressed by various cells including fibroblasts, macrophages, epithelial cells, endothelial cells, and astrocytes. Although IL-17RA has been detected on T cells, both primary cells and cell lines, exposure to IL-17A fails to induce the expression of the canonical IL-17 target genes in the absence of the RC receptor unit [7]. The main producers of IL-17A and IL-17F are Th17, as reflected by their signature cytokines, CD4/CD8 double-negative TCR*α*ß^+^ T cells (DNT; see later) and to a lesser degree cytotoxic CD8^+^ T cells (mucosal-associated invariant (MAIT) T cells, gamma-delta (*γδ*) T cells, and innate lymphoid cells like group 3 innate lymphoid cells (ILC3)) [2, 8].

In the last three decades, the immunological functions of IL-17A in the context of different clinical settings have been increasingly elucidated. A large pile of evidence alludes to IL-17A as first defence to preserve the barrier integrity of epithelial organs including skin and respiratory and gastrointestinal tracts to fight against invading pathogens, particularly extracellular bacteria and fungi. Clinical data from human models of defective IL-17A signaling, resulting from genetic, therapeutic, or viral causes, have underpinned its crucial role for the antimicrobial immunity surveillance across exposed surfaces [9–11]. Amongst the primary mechanisms of IL-17A to maintain epithelial barrier integrity is stimulating the production of antimicrobial peptides such as *β*-defensins and S100A8 (Calgranulin A) and S100A9 (Calgranulin B) that are together expressed as the heterodimer calprotectin. Further, IL-17A may prevent colonic injury and restore intestinal epithelial homeostasis by triggering the expression of tissue plasminogen activator (tPA), with subsequent activation of TGF-*β*-mediated anti-inflammatory pathways [12]. In the skin, IL-17A has been implicated in physiological wound repair by inducing the expression of regenerating islet-derived protein 3-alpha (REG3A) that promotes proliferation in keratinocytes [13]. Second, IL-17A attracts effector cells to the site of inflammation to help eliminate potential threats and assist in the repair of tissue damage. Illustratively, the IL-17A/G-CSF axis has been involved in regulation of bone marrow granulopoiesis and neutrophil recruitment to the inflammatory site. In this respect, counterregulatory mechanisms involving CXCR2 expression on neutrophils, CXCL5, and commensal bacteria are instated to keep neutrophil homeostasis in check [14]. Third, IL-17A in synergy with IL-13 (in a CXCL13-dependent manner) may support the host response to intracellular pathogens by driving the development of ectopic lymphoid structures, composed of highly organized T cell and B cell zones that emerge during infections with intracellular pathogens such as pneumocystis jiroveci and mycobacterium or in response to inflammatory stimuli [15, 16]. Several of these immune properties of IL-17A have been recapitulated in autoinflammatory disorders and more recently cancer, and on top of that, new features have been uncovered [17, 18]. As for the latter, IL-17A assists in shaping the tumor microenvironment by dampening tumor-specific immune responses involving proangiogenic signals, progressive loss of antitumor Th1 immunity, and suppression of T cell immune surveillance [19, 20]. In addition, IL-17A has been shown to promote protumorigenic factors like proliferation capacity, immune cell infiltration, resistance to chemotoxicity, and migratory and invasive properties [19–26].

## 3. IL-17A Signaling Pathway

At first sight, IL-17A represents a proinflammatory cytokine that appears to have only modest properties *in vitro* as compared to other cytokines such as TNF and IL-6. Thus, IL-17A signaling initiates a cascade of events that results in transcriptional regulation of a variety of inflammatory RNAs with the release of their corresponding proteins that are dominated by a set of signature genes comprising IL-6, granulocyte colony-stimulating factor (G-CSF), chemokines such as CXCL1-2, CCL20, lipocalin-2 (Lnc2), metalloproteinases, and beta defensins [27, 28]. Adapter molecule Act1 encoded by the gene TRAF3IP2 (also known as CIKS) mediates the downstream signaling of IL-17A and is therefore essential for its transcriptional and posttranslational mechanisms [28]. Act1, containing different TNF receptor-associated factor (TRAF) binding motifs, constitutes a multifunctional platform for various TRAFs, which context-dependently may be recruited to trigger different downstream pathways. Thus, the IL-17R/Act1 complex recruits the adaptor molecule TRAF6 as intermediate component in the signaling cascade, which subsequently results in TGF-*β*-activated kinase (TAK) 1 phosphorylation and feedforward activation of the canonical NFkB pathway but also the MAPK pathways p38, ERK, or JNK [2, 29]. IL-17A constitutes a weak activator of the NFkB signaling pathway, but instead, IL-17A signaling may activate other downstream targets including transcription factors CCAAT enhancer-binding protein (C/EBP-*β*), AP-1 complex, and I*κ*B*ζ* (encoded by NFKBIZ) [30]. Act1 can also recruit and interact with TRAF2 and TRAF5 and the splicing regulatory factor SF2 (ASF) to form a complex in order to prolong the stability of inflammatory mRNAs like CXCL1 mRNA (see further) [31]. Other TRAFs like TRAF4 may engage in antagonizing Act1-mediated induction of IL-17A-related inflammatory genes by competing with TRAF6 [32]. Further, Act-1 may suppress IL-23/IL-6-induced STAT3 inhibition as a negative regulator in T and B cells [33].

Engagement of IL-17R with Act1 is mediated via interaction between a “similar expression to fibroblast growth factor genes/IL17R” (SEFIR) domain in the cytoplasmic tail, which is a conserved region amongst all IL-17Rs, and the SEFIR domain present on Act1. The IL-17 target genes are enriched for DNA binding sites of the transcription factors (transcriptional regulatory elements) in their proximal promoter regions [27].

Other functions of Act1 include the E3 ubiquitin ligase activity towards TRAFs that may control their fate and activity in IL-17A signaling, as illustrated by the K63-linked polyubiquitination of TRAF6 mediated by TAK-1 [2, 34, 35]. Lysine-124 residue of TRAF6 has been implicated in Act1-mediated ubiquitination of TRAF6 and TRAF6's ability to mediate IL-17-induced activation of NFkB [36]. A counterregulatory mechanism is represented by the ubiquitin-specific peptidase 25, a deubiquitinating protease that reverses the modification of TRAF6 and thus decreases TRAF6 assembly to Act-1 by remodeling the K63 polyubiquitin.

In addition, Act1 has been found to suppress pathways mediated by CD40 and BAFF, both members of the TNF receptor (TNFR) superfamily, which play critical roles in B cell survival and differentiation. Thus, Act1 knockout mice develop lymphadenopathy and splenomegaly, hypergammaglobulinemia, and autoantibody formation, where Act1-deficient B cells exhibit stronger IkappaB (IkB) phosphorylation, NFkB2 signaling, and activation of JNK, ERK, and p38 pathways [37]. A similar phenotype was observed in IL-17RA knockout mice on a C57BL/6 *lpr* background [38].

Posttranscriptional regulation that may prolong or shorten RNA stability is another important feature of IL-17A signaling, a capacity that is mediated via mRNA-binding proteins that are seemingly at the crossroads of host immune response to microbial inflammation and the development of autoimmune disorders. RNA-binding proteins (RBPs), like AT-rich interactive domain-containing protein 5A (arid5a), act on the adenylate/uridylate- (AU-) rich elements (ARE) of the 3′untranslated region (3′UTR) for stabilizing various inflammatory mRNA genes and have specific target genes. Thus, Arid5a has been found to stabilize the IL-6 gene and not that of other cytokines like TNF, via competition with the ribonuclease (RNAse) Regnase I (see further) on the same region in 3′UTR of IL-6 [39, 40]. In addition, Arid5a promotes the stability of the mRNA of CXCL1 and CXCL5 amongst others. Other RBPs that can be recruited to the IL-17R/Act 1 complex via TRAF2 and TRAF5 include DEADbox helicase 3X-linked (DD3X3) and Hu-antigen R (HuR). The latter competes with RNA decay factor splicing factor 2SF2. Through selective stabilization of STAT3, Arid5a may skew differentiation of CD4^+^ T cells to the Th17 subset [41].

Noticeably, there are counterregulatory mechanism to constrain IL-17-induced inflammation. Regnase-1, also known as zinc finger CCCH-type containing 12A (ZC3H12A) or monocyte chemoattractant protein I-inducing protein (MCP1P1), represents an immune response modifier with RNase activity. Regnase-1 is a cytoplasm localized protein with a CCCH-type zinc finger motif that can be induced through toll-like receptor (TLR) signaling [42]. The RNase activity is mediated via a putative amino-terminal nuclease domain that sets off the decay of a set of inflammatory genes like IL-6, IL-12p40, and calcitonin receptor gene via their 3′UTR [42]. Mice lacking Regnase-1 display a phenotype of autoimmunity that resembles systemic lupus erythematosus (SLE) in humans, including antinuclear antibodies, anti-double-stranded DNA (dsDNA), autoantibodies, hyperglobulinemia, anemia, plasma cell infiltration in lung interstitial tissue, and splenomegaly and lymphadenopathy [42]. A similar phenotype has been found in the lupus-prone Sanroque mice, where a key role for another RING-type ubiquitin ligase protein with a CCCH-type zinc-finger domain Roquin has been established in repressing autoimmunity [43]. Recently, Regnase-1 has been identified as negative regulator of antitumor activity of CD8^+^ T cells and thereby suppressing their accumulation and mitochondrial fitness by targeting BATF (rheostat) [44]. A recent study has pointed to IL-17-mediated Act1/DDX3X interaction that controls stability of Regnase-1 [45].

Negative inhibitors of IL-17A signaling involve the deubiquitinase zinc-finger protein A20, a key player in the negative feedback regulation of NF-*κ*B pathway, which is mediated via the CEBP beta activity domain (CBAD) [46]. Other negative regulators of IL-17A signaling includes noncoding RNA miR-23B that targets TAB2 and TAB3 and miR30a that induces degradation of Act1 [47, 48]. In certain conditions of inflammation, IL-17A may team up with other proinflammatory mediators like epidermal growth factor, FGF2, and Notch1 [18, 49, 50]. Further, synergistic activities of TNF and IL-17A, involving transcription factors CUX1 and I*κ*B*ζ* (NFKBIZ), have been described in stromal-resident fibroblast-like synoviocytes, resulting in secretion of IL-6 and CXCL8 and neutrophil recruitment [51].

## 4. The Role of IL-17A in Systemic Lupus Erythematosus

SLE represents a heterogeneous, multicompartment autoimmune disease that may involve the skin, lymphatic network, musculoskeletal system, and internal organs like kidney, lungs, and central nervous system (CNS) [52]. Next to clinical manifestations, SLE features various serological abnormalities including autoantibody formation, hypergammaglobulinemia, hypocomplementemia, and autoimmune-mediated cytopenias. Prototypically, the antinuclear antibodies consist of (1) anti-nucleosome autoantibodies that are directed at DNA, histones, or DNA-histone complex, (2) cytoplasmic proteins, (3) RNA, or (4) U1-small nuclear ribonucleoprotein complex like U1-70 [53]. SLE is associated with long-term morbidity, coexistential disorders like cardiovascular disease, and an increased risk of death.

To date, several studies in young and adult SLE patients have reported on the association of increased serum IL-17A levels or frequencies of IL-17A expressing T cells with disease severity, particularly in those with CNS involvement [52, 54–61]. In this regard, good interpretation of these studies has been flawed by the limited numbers of patients, heterogeneity of disease manifestations, and a large proportion of study subjects on various treatment strategies. Preclinical studies that have hinted at involvement of IL-17A in SLE pathology indicate that the absence of IL-17 in experimental lupus models is associated with inhibition of autoantibody formation targeting DNA, RNP, and chromatin and, even more striking, lupus nephritis [62, 63].

A closer look at the T cell compartment, which exhibits various anomalies in cytokine production and cellular functions in active SLE, may offer insights into the mechanisms underlying these associations. Thus, patients with active SLE display a marked expansion of IL-17A-expressing T cell subpopulations comprising CD4^+^ Th17 cells and DNTs [57, 64, 65]. Normally, DNTs take up 1% to 2% of the total T cell pool in peripheral blood and lymph nodes (LN) of healthy donors [66]. These IL-17A^+^ subpopulations are antigen specific, given that tetramer studies have identified ROR*γ*t^+^ IL-17A-producing T cells that are specific for U1-70 in humans and lupus-prone mice [67]. Similar to Th17 cells, DNTs express ROR*γ*t, the master regulator of the Th17 lineage, and IL-23R [57, 64]. In lupus-prone mice, IL-23 has been identified as an important driver of DNT expansion and IL-17A production [68]. Phenotypically, peripheral DNTs exhibit extraordinary migratory and tissue invasive properties, which may result in severe organ pathology in various inflammatory settings like ischemic stroke and spondyloartropathy [57, 69, 64, 70, 71]. Their origin is not completely understood; however, earlier studies have suggested that DNTs originate from the thymus and spleen. Recently, splenic marginal zone macrophages (MZMs) have been implicated as regulators of DNT development. Upon experimental depletion of MZMs, the compartment of autoreactive CD8^+^ T cells expand and lose their CD8 expression to adopt the DNT phenotype including loss of regulatory properties, enhanced migratory potential, IL-17A-producing potency, hyperproliferative state, and a narrowed TCR repertoire [72]. Another regulator of DNT development that has been suggested is Act1 [33]. The expansion of splenic DNTs instigates hallmark symptoms of SLE in lupus-prone mice including the emergence of germinal centers (GC), generation of anti-double-stranded DNA (dsDNA) autoantibodies, and inflamed kidney characterized by infiltration of DNTs [72].

**Figure 2:**
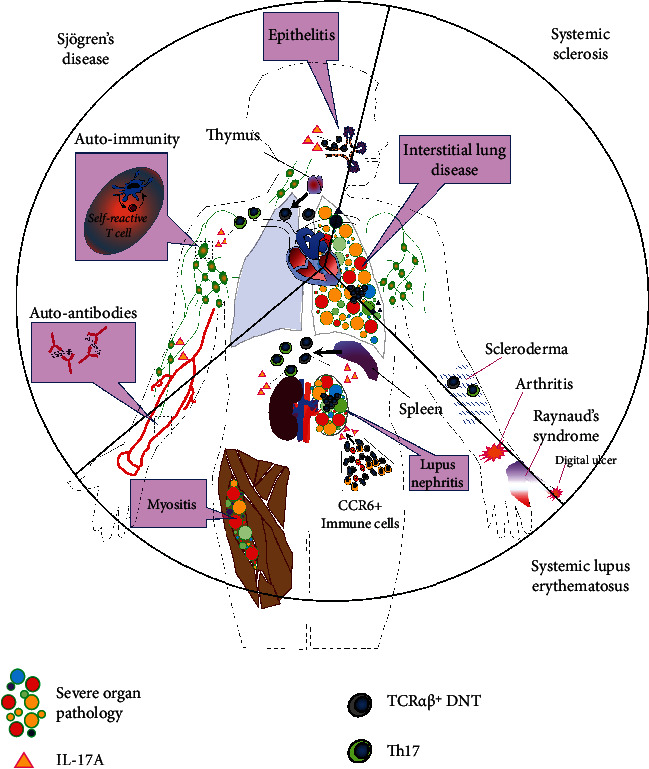
Schematic representation of the contributive role of IL-17A in the pathogenesis of three major systemic autoimmune diseases. Growing evidence suggests that the abundance of IL-17A and its prime source, i.e., Th17 cells and DNTs, in the target tissues may deteriorate clinical and immunological patterns in any of these autoimmune disorders by promoting (1) autoimmunity, immune cell recruitment, and vascular-immune interactions in SLE, (2) induction of autoantigen expression and undermining of endothelial integrity/barrier function in SS, and (3) fibrogenesis (indirect mechanism) in myofibroblast precursors and vasculopathy in SSC. Th17 and DNTs, originating from the spleen and thymus, display excellent properties to infiltrate disease-associated organs, which in concert with tissue-derived factors ensure coordinated temporal-spatial distribution as well as activation of IL-17A-expressing T cells within lymphoid and nonlymphoid tissues.

Apart from the peripheral blood compartment, IL-17A-expressing T cells have been detected in various SLE-related target tissues. Thus, IL-17A-producing cells particularly DNTs have been shown to invade inflamed kidneys of lupus nephritis patients (see Figure 2) [64]. In more detail, infiltrating IL-17A-expressing T cells gather in close proximity to blood, which has been shown in vascular beds of skin and lungs [57]. In pediatric SLE patients with pulmonary involvement, these IL-17A^+^ T cells have been postulated to exert direct adverse effects on airway smooth muscle remodeling worsening small airway obstruction [59, 73]. Their presence seems to be a prerequisite for organ pathology SLE. Thus, regression of DNT presence in the lupus-prone B6/lpr mice, as mediated by IL-23R deficiency, was associated with mitigation of lymphoid hyperplasia and suppression of the development of lupus nephritis [74].

Based on the available evidence from preclinical mouse models, the contribution of IL-17A to the disease pathology in SLE appears to be reflected by four different mechanisms.

First, IL-17A mediates the recruitment of effector cells like neutrophils, IL-17A^+^-expressing T cell subsets, and CCR6^+^ B cells into SLE target tissues and GCs [75]. Second, IL-17A may represent a driving force behind autoimmunity [76]. In the BXD2 mouse model that recapitulates many SLE features like enhanced activation-induced cytidine deaminase (AICDA) activity, autoantibody generation, circulating immune complexes, and progressive glomerulonephritis, IL-17A has been shown to induce and stabilize autoreactive GC formation via B cell retention within GCs and increased CXCL12/CXCR4-mediated interactions between B cells and T cells resulting in AICDA upregulation and autoantibody generation [77–79]. Further, IL-17A may promote class switching to IgG2a and IgG3, plasma cell development, and MHC class II expression on B cells, whereas DNT derived from patients with SLE have been found to directly promote cationic IgG antibodies against DNA in coculture [78–80].

Third, IL-17A may play a role in enhanced vascular-immune interactions. Thus, endothelial activation upon exposure to IL-17A derived from PBMC of patients with active SLE promotes the adherence of Jurkat cells to vascular endothelium, which is mediated by augmented endothelial expression of E-cadherin, ICAM-1, and VCAM-1 [57]. The last mechanism refers to the T cell-neutrophil interaction as partners in crime. The release of neutrophil extracellular trap (NET) formation has been implicated in the pathogenesis and organ injury in SLE, which is driven by increased REDD1/autophagy axis [81]. Interestingly, depositions of NET in actively inflamed skin and kidney have been found to colocalize with bioactive tissue factor and IL-17A that in a synergistic manner may promote fibrotic activity in the stromal cell compartment [81]. In Fc gamma receptor IIb- (Fcgr2b-) deficient mice that develop fatal lupus pathology, IL-17A/Act1 signaling has been shown to adversely affect the course of glomerulonephritis by promoting the recruitment of immune cells in particular neutrophils and NET deposition in inflamed kidneys [79].

The external and intrinsic factors that enable Th17 and DNT subpopulations to successfully invade target tissues and promote SLE pathogenesis are ample. Aside from the inflammatory microenvironmental milieu that is enriched with chemoattractants like CCL20, endothelium-derived CD95 expression has been shown to promote infiltration of IL-17A-expressing T cells into the perivascular space in a PI3K- and calcium signaling-dependent manner [82]. The chemokine receptor CCR6, a nonpromiscuous receptor with as sole ligand C-C motif chemokine ligand 20 (CCL20), has been found to a play a key role in the trafficking of Th17 cells to the inflamed kidney in experimental lupus nephritis [83]. In this respect, the serine/threonine kinase calcium/calmodulin-dependent kinase IV may promote CCR6 expression in IL-17A-expressing T cells, as well as CCL20 secretion that recruits other CCR6^+^ T cells through a positive feedback mechanism that may propagate tissue inflammation and accelerate glomerular injury in the inflamed kidney [60]. In children with lupus nephritis, enhanced migratory activities of IL-17A-expressing T cell subsets have been linked to enhanced Akt signaling [61]. Moreover, expansion of IL-17A-expressing T cells has been ascribed to heightened intrinsic activity of the nonreceptor phosphatase (PTP) protein tyrosine phosphatase SH2 domain-containing PTP (SHP2) in humans and mice [84]. Using adoptive transfer studies, fate reporter mice, and mouse models of lupus nephritis, kidney-infiltrating Th17 cells have been found to display very limited spontaneous plasticity, where Th17 cells usually show high degree of plasticity to transdifferentiate into other T cell phenotypes upon inflammatory stimuli [85]. Last, SLE may be associated with functional impairment of CD147 (basigin), an extracellular matrix metalloproteinase inducer (EMMPRIN), which may act as a brake on the disproportional expansion of Th17 cells [86].

Enhanced activity of IL-17A-expressing T cell subsets has been ascribed to increased ROCK activity [87, 88]. In addition, SLE T cells exhibit augmented expression of signaling lymphocyte activation molecules (SLAMs). Particularly, expression of SLAMF6 and 3 has been associated with superior costimulatory activity *in vitro*, as compared to CD3/CD28 [89, 90]. Inversely, SLAMF1 ligation in cocultures of B and T cells may reduce IL-17A and IL-21 production [91]. Another mechanism involves synergistic activity between enhanced recruitment of ROR*γ*t to the IL-17A promoter and CD28-induced nuclear abundance of the transcription factor nuclear factor of activated T cells (NFAT) [92]. The latter can be dampened by dipyridamole, a recently recognized specific inhibitor of calcineurin–NFAT interactions [93]. Further, upregulated expression of ubiquitin-specific protease 17 (USP17) in CD4^+^ T cells from SLE patients has been found to prolong RORyt-dependent IL-17A transcription by increasing the stability of RORyt and preventing it from proteosomal degradation [94]. Another modifier that has been identified to augment IL-17A production is transcription factor friend leukemia integration 1 (Fli-1) that regulates the expression of numerous cytokines and chemokines [95]. Last, epigenetic mechanisms that have been implicated in SLE pathogenesis involve cAMP response modulator (CREM)*α* that mediates demethylation of IL-17A promoter and trans-repression of the IL-2 gene, resulting in enrichment of effector memory T cell phenotypes [65]. In juvenile onset lupus, CREM*α* has been recognized to drive increased IL-17A expression and reduced IL-2 production in CD4^+^ T cells [96].

## 5. The Role of IL-17A in Sjögren's Syndrome

The concept for the extent to which IL-17A is involved in the pathogenesis of SS is less developed as compared to SLE, given the restricted amount of clinical and experimental data. SS presents itself primarily with sicca syndrome and exocrine gland dysfunction that results from lymphocytic infiltration into lacrimal and salivary glands (SG). In addition, SS features a constellation of clinical and serological signs, consisting of autoantibody formation, hypergammaglobulinemia, fatigue, arthritis, cutaneous manifestations, and increased risk for malignant lymphomas [97]. The prototypical autoantibodies target Ro/SSa (two subunits, 52 kDa and 60 kDa) and La/SSb antigens and may be detected up to ~5 years before diagnosis [98, 99]. Ro52 constitutes an E3 ligase that belongs to the tripartite motif family and has been implicated in the transcriptional regulation of proinflammatory cytokines like IL-17A given its RING-dependent E3 ligase activity [100]. Also, Ro52 regulates several members of the interferon regulatory factor (IRF) family like IRF3 that suppress IL-17A and IL-23R expression by holding off ROR*γ*t from accessing corresponding DNA-binding sites/enhancer regions [101, 102]. In a Ro52 reporter (Rho-deficient) mouse strain, where the Ro52 locus is replaced by GFP, tissue-specific enrichment of Ro52 protein expression is detectable in lymphoid tissues, including spleen, LNs, and thymus, which corresponds with the clinical picture of SS ^100^. Ro60 represents a RNA-binding protein that has been implicated in environmental stress, and its loss has been associated with photosensitivity and cutaneous lesions in SLE [103].

Several exploratory studies in SS patients and preclinical mouse models have reported on the association between IL-17A and SS pathology, including increased IL-17A levels in serum and in target tissue SGs and the lacrimal system [104–110]. Moreover, histological examination of SGs of patients, suffering from SS, reveals a lymphocytic infiltration, the majority of which are IL-17A-expressing CD4^+^ T cells and to lesser degree CD8^+^ T cells (see Figure 2) [111].

Additional confirmation for IL-17A involvement in SS comes from observations in the Ro52-null mice that develop SS-like manifestations comprising dermatitis, autoantibody formation, hypergammaglobulinemia, lymphadenopathy, splenomegaly, and kidney pathology characterized by proteinuria with mesangium and intraglomerular immunoglobulin depositions [100]. Tissue inflammation and the overactive immune system in these mice display an “IL-17A signature,” as attested by hyperproliferating LN and spleen cells that spontaneously secrete IL-17A (and related cytokines), which together with a substantial enrichment for IL-17A-expressing T cells in the CD4^+^ and CD4^−^ compartments could be jacked up by T cell activation. Conversely, abrogation of the IL-23/IL-17A axis in these Rho52-null mice restores a substantial part of the SS-related pathology [100]. Of note, the effects of IL-17 ablation on SS-like manifestations appear to be more prominent in female animals, suggesting sexual dimorphism [110]. Other lines of evidence that implicates the IL-17A pathway in the pathogenesis of SS derives from adenovirus-mediated delivery studies (IL-17A overexpression) and genetically engineered mouse models (IL-17A entrapment). Thus, adenovirus-induced IL-17A overexpression in SGs of nonsusceptible C57Bl/6 gives rise to pathognomonic signs of SS that include decreased saliva production, lymphocytic infiltration in SGs, and positive ANA test with a fine nuclear speckled pattern [112]. Inversely, IL-17A entrapment through a fusion protein that combines IL-17R and a Fc portion (IL-17R:Fc) results in amelioration of the clinical and immunological pattern in established mouse models for SS [109, 113, 114].

Based on currently available data, the contribution of IL-17A to the pathophysiology of SS is reflected by three different mechanisms. First, IL-17A may induce the expression of autoantigens, characteristically that of glandular tissue kallikreins (KLK) which belong to the large KLK family of serine proteases. Thus, glandular KLK13, which is found to be enriched in striated duct cells of SGs ^115^, shows enhanced expression in SS-like IQI/Jic mice and acts as proliferative stimulus for splenic T cells [116]. Moreover, SG-derived KLK13 and KLK1 exhibit cross-reactivity with autoantibodies in serum of IQI/Jic mice [116]. In a similar manner, KLK1b22 has been found to be upregulated in the SGs of SS-like ERdj5 knockout mice [117]. Noticeably, proteomic analysis of glandular tissues in SS-like Aec1/Aec2 mice that underwent ultrasound-guided adenoviral-mediated IL-17R:Fc gene therapy of the SGs reveals that IL-17A entrapment is associated with reduced expression of KLK1b22 [118].

Second, IL-17A has been implicated in the impairment of the epithelial tight junction (TJ) integrity and barrier function of SGs. In more detail, IL-17A appears to mediate SG tissue damage and salivary dysfunction in NOD and Aec1/Aec2 mice by targeting Claudin-4 and zonula occludens I, both functional and structural components that are crucial to TJs [110, 119].

Third, IL-17A may promote an inflammatory environment within target tissues like SGs through IL-6 expression [111] that may facilitate mononuclear recruitment and infiltration [115] and invigorate the Th17 differentiation program.

DNTs, as in SLE, may play a contributive role in the pathogenesis of SS, given their expansion in the peripheral blood and SG compartments of SS patients (see Figure 2) [70]. Of note, mast cells are considered a potential source for IL-17A, as their numbers, in parallel to IL-17A expression, shrink in SS patients in response to anti-CD20 therapy [120].

Tissue-derived factors that may regulate IL-17A expression in CD4^−^ and CD4^+^ T cell compartments include IL-27. Thus, IL-27 display an inhibitory effect on IL-17A secretion in PBMC cultures of SS patients and not RA patients or age-matched healthy donors [121]. Further, induction of experimental sialadenitis in IL-27receptor subunit alpha knockout mice aggravates the formation of ectopic lymphoid structures in SGs, as compared to wild-type mice, a finding that can be restored by IL-17A neutralization [121]. Other local mediators that may control IL-17 expression are the lysophosphatidic acid receptor signaling pathway and retinoic ROR*α* that together with ROR*γ*t and I*κ*B*ζ* may promote IL-17A transcription and Th17 differentiation [122, 123].

## 6. The Role of IL-17A in Systemic Sclerosis

SSC is a multiorgan connective tissue disease that is characterized by high morbidity and mortality related to organ complications like lung fibrosis and pulmonary arterial hypertension [124]. Approximately 1 in 10,000 people appears to be affected globally.

The triad of pathologic changes that defines SSC comprises autoimmunity, vasculopathy, and fibrosis of skin and internal organs. Fibrosis, a hallmark of more advanced SSC disease stages, results from excess deposition of extracellular matrix (ECM) and differentiation of mesenchymal stromal cells like fibroblasts and endothelial cells into myofibroblasts, a key determinant of end-stage SSC pathology [125, 126]. The fibrosis stage in SSC is preceded by an edematous phase that is characterized by mononuclear cell infiltrates in the dermis, comprising plasma cells, IL-13-producing CD8^+^ T cells, and Th17 [127, 128], and progressive failure of the locoregional blood and lymphatic vasculature [124]. Consecutively, these events may result in lymphedema with ensuing elastin degeneration, hyperplasia/hypertrophy of adipose tissue, and increase of collagen fibres and fibrous deposits that causes hardening of the skin.

Several association studies in humans and experimental mouse models have hinted at the contributive role of the IL-17A pathway in the pathogenesis of SSC, showcasing increased IL-17A levels (or IL-23 as partner in crime) in serum and affected skin, as well as increased frequencies of IL-17A^+^-producing T cells(see Figure 2) [126, 129–133, 125, 134]. In the SSC skin, IL-17A-expressing T cells have been detected throughout the skin, both superficial and deep layers and in close proximity of *α*SMA-positive myofibroblasts [134]. In these studies, enhanced IL-17A production has been repeatedly associated with early stages of SSC [131, 135]. Outside the skin, augmented IL-17A expression is detectable in lymphocytes, derived from peripheral blood and bronchoalveolar lavage of SSC patients. Involvement of the IL-17A pathway in SSC has been further suggested by a large case-control study involving three different European populations, where polymophisms in the chemokine receptor CCR6 gene have been associated with increased susceptibility to SSC, particularly in patients with an anti-topoisomerase (Scl-70) autoantibody profile [136].

The implication of IL-17A in the pathophysiology of SSC has been fueled by several findings associated with disease progression. The mechanism that has gained increasing attention involves the impact of IL-17A on the fibroblast phenotype and its transition to myofibroblasts. According to findings in two established SSC-like mouse models, the contributive role of IL-17A in the progression of skin fibrosis is reflected by enhanced leucocyte recruitment and a driving force behind the expression of the profibrotic mediators transforming growth factor (TGF) *β* and connective tissue growth factor (CTGF) in the skin [125]. In addition, these authors and others reveal the capacity of IL-17A to stimulate collagen production in cultured mouse and human skin fibroblasts [125, 126]. However, the concept that IL-17A directly mediates fibrogenesis in the SSC dermal fibroblast has become controversial after conflicting data from more recent work. These studies demonstrate a stimulatory effect on the proliferating response in SSC and control skin fibroblasts, but fail to show enhanced collagen synthesis whether or not in the presence of TGF*β* [135, 137, 132, 138, 139]. The discrepancy in results between these studies may be explained by different experimental settings, as well as distinct methods of fibroblast isolation and culture.

Second, IL-17A may assist in shaping the inflammatory milieu within target tissues, through enhancement of immune cell recruitment, tissue migration, and vascular immune interactions via cytokines (e.g., MCP-1, IL-6, and IL-8), chemokine networks (CCL20-CCR6, CXCL12-CXCR4), endothelial adhesion molecules, and metalloproteinases [137, 134, 130, 132, 135]. Synergistic activity of IL-17A and TGF*β* in terms of inducing the expression of these inflammatory cues has been reported [138]. A third way involves the negative impact of IL-17A on SSC-associated vasculopathy. In this regard, IL-17A has been shown to exert adverse effects on dermal vascular smooth muscle cells (DVSMCs) that may promote vascular wall fibrosis and microangiopathy [140]. Analogue to pathological changes of the blood vasculature in the skin, progressive loss of lymphatic vessels (rarefaction) has been acknowledged, particularly in the advanced stages of SSC [141]. Besides mechanisms that involve anti-endothelial cell autoantibodies and dysregulated expression of vascular growth factors, IL-17A and TNF are amongst the candidate cytokines that may (potentially in concert) negatively affect lymphatic neovascularization [142, 143].

Like the aforementioned disorders, SSC has been associated with increased numbers of DNTs in peripheral blood, where particularly the V*α* and V*β* repertoires seem narrowed in diversity, as compared to CD4^+^ and CD8^+^ T cells [144, 145]. Moreover, these T cell subsets with restricted usage of TCR V*β* genes tend to oligoclonally expand in the SSC skin (rather than in the peripheral blood compartment), suggesting an autoantigen-driven process, and this phenomenon ceases in later stages of the disease [146, 147]. Additional anomalies in the peripheral T cell pool that may promote IL-17A expression involve the emergence of FoxP3^+^IL17^+^ T cells with reduced suppressive capacity and higher RORC expression in the regulatory T cell compartment, which may point to increased Treg-to-Th17 transition [148].

Local tissue-derived factors that may promote IL-17A-expressing T cells involve the inducible T cell costimulator- (ICOS-) ICOSL axis. Thus, SS patients, particularly in early stages of SSC, show higher levels of sICOS in serum and increased ICOSL expression in lesional skin, where ICOS costimulation induces the expression of IFN*γ* and IL-17A as well as profibrogenic cytokines (IL-4) from CD4^+^ T cells [149].

## 7. Conclusive Remarks

In conclusion, this review presents evidence of the ability of IL-17A to drive the development and exacerbation of clinical manifestations in three major autoimmune diseases. Available studies provide convincing evidence that the abundance of IL-17A and its prime source, i.e., Th17 cells and DNTs in the target tissues, is not an innocent bystander but in fact seems to be prerequisite for organ pathology. In support, IL-17A has been directly implicated in critical steps of autoimmunity comprising the emergence and stabilization of autoreactive GC formation, AICDA upregulation, class switching to IgG2a and IgG3, and autoantibody generation. In addition, this review reports on the synergistic interactions of IL-17A with other critical determinants such as B cells, neutrophils, stromal cells, and the vasculature that promote the characteristic immunopathology of these autoimmune diseases. The summary of observations provided by this review may have empowering implications for IL-17A-based strategies to prevent clinical manifestations in a broad spectrum of autoimmune conditions.

## References

[1] Gaffen S. L. (2009). Structure and signalling in the IL-17 receptor family. *Nature Reviews Immunology*.

[2] Li X., Bechara R., Zhao J., McGeachy M. J., Gaffen S. L. (2019). IL-17 receptor-based signaling and implications for disease. *Nature Immunology*.

[3] Rouvier E., Luciani M. F., Mattei M. G., Denizot F., Golstein P. (1993). CTLA-8, cloned from an activated T cell, bearing AU-rich messenger RNA instability sequences, and homologous to a herpesvirus saimiri gene. *The Journal of Immunology*.

[4] Yao Z., Fanslow W. C., Seldin M. F. (1995). Herpesvirus Saimiri encodes a new cytokine, IL-17, which binds to a novel cytokine receptor. *Immunity*.

[5] Huang J., Lee H. Y., Zhao X. (2021). Interleukin-17D regulates group 3 innate lymphoid cell function through its receptor CD93. *Immunity*.

[6] Su Y., Huang J., Zhao X. (2019). Interleukin-17 receptor D constitutes an alternative receptor for interleukin-17A important in psoriasis-like skin inflammation. *Science immunology*.

[7] Lindemann M. J., Hu Z., Benczik M., Liu K. D., Gaffen S. L. (2008). Differential regulation of the IL-17 receptor by gammac cytokines: inhibitory signaling by the phosphatidylinositol 3-kinase pathway. *Journal of Biological Chemistry*.

[8] Cai Y., Shen X., Ding C. (2011). Pivotal role of dermal IL-17-producing gammadelta T cells in skin inflammation. *Immunity*.

[9] Brenchley J. M., Paiardini M., Knox K. S. (2008). Differential Th17 CD4 T-cell depletion in pathogenic and nonpathogenic lentiviral infections. *Blood*.

[10] Milner J. D., Brenchley J. M., Laurence A. (2008). Impaired T (H)17 cell differentiation in subjects with autosomal dominant hyper-IgE syndrome. *Nature*.

[11] Deodhar A., Mease P. J., McInnes I. B. (2019). Long-term safety of secukinumab in patients with moderate-to-severe plaque psoriasis, psoriatic arthritis, and ankylosing spondylitis: integrated pooled clinical trial and post-marketing surveillance data. *Arthritis research & therapy*.

[12] Kaiko G. E., Chen F., Lai C. W. (2019). PAI-1 augments mucosal damage in colitis. *Science translational medicine*.

[13] Lai Y., Li D., Li C. (2012). The antimicrobial protein REG3A regulates keratinocyte proliferation and differentiation after skin injury. *Immunity*.

[14] Mei J., Liu Y., Dai N. (2012). Cxcr2 and Cxcl5 regulate the IL-17/G-CSF axis and neutrophil homeostasis in mice. *The Journal of clinical investigation*.

[15] Wang Z. Z., Song J., Wang H. (2020). Stromal cells and B cells orchestrate ectopic lymphoid tissue formation in nasal polyps. *Allergy*.

[16] Eddens T., Elsegeiny W., Garcia-Hernadez M. L. (2017). _Pneumocystis_ -Driven Inducible Bronchus-Associated Lymphoid Tissue Formation Requires Th2 and Th17 Immunity. *Cell reports*.

[17] Liu C., Liu R., Wang B. (2021). Blocking IL-17A enhances tumor response to anti-PD-1 immunotherapy in microsatellite stable colorectal cancer. *Journal for immunotherapy of cancer*.

[18] Chen X., Cai G., Liu C. (2019). IL-17R-EGFR axis links wound healing to tumorigenesis in Lrig 1(+) stem cells. *Journal of Experimental Medicine*.

[19] Dawod B., Liu J., Gebremeskel S. (2020). Myeloid-derived suppressor cell depletion therapy targets IL-17A-expressing mammary carcinomas. *Scientific reports*.

[20] Ma Y. F., Chen C., Li D. (2017). Targeting of interleukin (IL)-17A inhibits PDL1 expression in tumor cells and induces anticancer immunity in an estrogen receptor-negative murine model of breast cancer. *Oncotarget*.

[21] Benevides L., da Fonseca D. M., Donate P. B. (2015). IL17 promotes mammary tumor progression by changing the behavior of tumor cells and eliciting tumorigenic neutrophils recruitment. *Cancer research*.

[22] He D., Li H., Yusuf N. (2010). IL-17 promotes tumor development through the induction of tumor promoting microenvironments at tumor sites and myeloid-derived suppressor cells. *The Journal of Immunology*.

[23] Huang L. H., Zinselmeyer B. H., Chang C. H. (2018). Interleukin-17 drives interstitial entrapment of tissue lipoproteins in experimental psoriasis. *Cell Metabolism*.

[24] Cochaud S., Giustiniani J., Thomas C. (2013). IL-17A is produced by breast cancer TILs and promotes chemoresistance and proliferation through ERK1/2. *Scientific reports*.

[25] Wu S., Rhee K. J., Albesiano E. (2009). A human colonic commensal promotes colon tumorigenesis via activation of T helper type 17 T cell responses. *Nature medicine*.

[26] Chae W. J., Gibson T. F., Zelterman D., Hao L., Henegariu O., Bothwell A. L. (2010). Ablation of IL-17A abrogates progression of spontaneous intestinal tumorigenesis. *Proceedings of the National Academy of Sciences*.

[27] Shen F., Hu Z., Goswami J., Gaffen S. L. (2006). Identification of common transcriptional regulatory elements in interleukin-17 target genes. *Journal of Biological Chemistry*.

[28] Qian Y., Liu C., Hartupee J. (2007). The adaptor Act 1 is required for interleukin 17-dependent signaling associated with autoimmune and inflammatory disease. *Nature immunology*.

[29] Schwandner R., Yamaguchi K., Cao Z. (2000). Requirement of tumor necrosis factor receptor-associated factor (TRAF)6 in interleukin 17 signal transduction. *The Journal of experimental medicine*.

[30] McGeachy M. J., Cua D. J., Gaffen S. L. (2019). The IL-17 family of cytokines in health and disease. *Immunity*.

[31] Sun D., Novotny M., Bulek K., Liu C., Li X., Hamilton T. (2011). Treatment with IL-17 prolongs the half-life of chemokine CXCL1 mRNA via the adaptor TRAF5 and the splicing-regulatory factor SF2 (ASF). *Nature immunology*.

[32] Zepp J. A., Liu C., Qian W. (2012). Cutting edge: TNF receptor-associated factor 4 restricts IL-17-mediated pathology and signaling processes. *The Journal of Immunology*.

[33] Zhang C. J., Wang C., Jiang M. (2018). Act 1 is a negative regulator in T and B cells via direct inhibition of STAT3. *Nature immunology*.

[34] Talreja J., Samavati L. (2018). K63-linked polyubiquitination on TRAF6 regulates LPS-mediated MAPK activation, cytokine production, and bacterial clearance in toll-like receptor 7/8 primed murine macrophages. *Frontiers in immunology*.

[35] Garcia-Barcena C., Osinalde N., Ramirez J., Mayor U. (2020). How to inactivate human ubiquitin E3 ligases by mutation. *Frontiers in Cell and Developmental Biology*.

[36] Liu C., Qian W., Qian Y. (2009). Act1, a U-box E3 ubiquitin ligase for IL-17 signaling. *Science Signaling*.

[37] Qian Y., Qin J., Cui G. (2004). Act1, a negative regulator in CD40- and BAFF-mediated B cell survival. *Immunity*.

[38] Corneth O. B. J., Schaper F., Luk F. (2019). Lack of IL-17 receptor A signaling aggravates lymphoproliferation in C57BL/6 lpr mice. *Scientific Reports*.

[39] Masuda K., Ripley B., Nishimura R. (2013). Arid 5a controls IL-6 mRNA stability, which contributes to elevation of IL-6 level in vivo. *Proceedings of the National Academy of Sciences*.

[40] Masuda K., Kimura A., Hanieh H. (2011). Aryl hydrocarbon receptor negatively regulates LPS-induced IL-6 production through suppression of histamine production in macrophages. *International Immunology*.

[41] Masuda K., Ripley B., Nyati K. K. (2016). Arid5a regulates naive CD4+ T cell fate through selective stabilization of Stat3 mRNA. *Journal of Experimental Medicine*.

[42] Matsushita K., Takeuchi O., Standley D. M. (2009). Zc3h12a is an RNase essential for controlling immune responses by regulating mRNA decay. *Nature*.

[43] Vinuesa C. G., Cook M. C., Angelucci C. (2005). A RING-type ubiquitin ligase family member required to repress follicular helper T cells and autoimmunity. *Nature*.

[44] Wei J., Long L., Zheng W. (2019). Targeting REGNASE-1 programs long-lived effector T cells for cancer therapy. *Nature*.

[45] Somma D., Mastrovito P., Grieco M. (2015). CIKS/DDX3X interaction controls the stability of the Zc3h12a mRNA induced by IL-17. *The Journal of Immunology*.

[46] Garg A. V., Ahmed M., Vallejo A. N., Ma A., Gaffen S. L. (2013). The deubiquitinase A20 mediates feedback inhibition of interleukin-17 receptor signaling. *Science Signaling*.

[47] Wan Q., Zhou Z., Ding S., He J. (2015). The miR-30a negatively regulates IL-17-mediated signal transduction by targeting Traf3ip2. *Journal of Interferon & Cytokine Research*.

[48] Zhu S., Pan W., Song X. (2012). The microRNA miR-23b suppresses IL-17-associated autoimmune inflammation by targeting TAB2, TAB3 and IKK-alpha. *Nature medicine*.

[49] Shao X., Chen S., Yang D. (2017). FGF2 cooperates with IL-17 to promote autoimmune inflammation. *Scientific Reports*.

[50] Wang C., Zhang C. J., Martin B. N. (2017). IL-17 induced NOTCH1 activation in oligodendrocyte progenitor cells enhances proliferation and inflammatory gene expression. *Nature Communications*.

[51] Slowikowski K., Nguyen H. N., Noss E. H. (2020). CUX1 and IkappaBzeta (NFKBIZ) mediate the synergistic inflammatory response to TNF and IL-17A in stromal fibroblasts. *Proceedings of the National Academy of Sciences*.

[52] Tsokos G. C. (2011). Systemic lupus erythematosus. *New England Journal of Medicine*.

[53] Pisetsky D. S., Lipsky P. E. (2020). New insights into the role of antinuclear antibodies in systemic lupus erythematosus. *Nature Reviews Rheumatology*.

[54] Vincent F. B., Northcott M., Hoi A., Mackay F., Morand E. F. (2013). Clinical associations of serum interleukin-17 in systemic lupus erythematosus. *Arthritis Research & Therapy*.

[55] Wong C. K., Ho C. Y., Li E. K., Lam C. W. (2000). Elevation of proinflammatory cytokine (IL-18, IL-17, IL-12) and Th2 cytokine (IL-4) concentrations in patients with systemic lupus erythematosus. *Lupus*.

[56] Talaat R. M., Mohamed S. F., Bassyouni I. H., Raouf A. A. (2015). Th1/Th2/Th17/Treg cytokine imbalance in systemic lupus erythematosus (SLE) patients: correlation with disease activity. *Cytokine*.

[57] Yang J., Chu Y., Yang X. (2009). Th17 and natural Treg cell population dynamics in systemic lupus erythematosus. *Arthritis & Rheumatism*.

[58] Edelbauer M., Kshirsagar S., Riedl M. (2012). Activity of childhood lupus nephritis is linked to altered T cell and cytokine homeostasis. *Journal of Clinical Immunology*.

[59] Hammad A., Osman E., Mosaad Y., Wahba M. (2017). Serum interleukin-17 in Egyptian children with systemic lupus erythematosus: is it related to pulmonary affection?. *Lupus*.

[60] Koga T., Otomo K., Mizui M. (2016). Calcium/calmodulin-dependent kinase IV facilitates the recruitment of interleukin-17-producing cells to target organs through the CCR6/CCL20 axis in Th17 cell-driven inflammatory diseases. *Arthritis & Rheumatology*.

[61] Kshirsagar S., Riedl M., Billing H. (2014). Akt-dependent enhanced migratory capacity of Th17 cells from children with lupus nephritis. *The Journal of Immunology*.

[62] Amarilyo G., Lourenco E. V., Shi F. D., La Cava A. (2014). IL-17 promotes murine lupus. *The Journal of Immunology*.

[63] Summers S. A., Odobasic D., Khouri M. B. (2014). Endogenous interleukin (IL)-17A promotes pristane-induced systemic autoimmunity and lupus nephritis induced by pristane. *Clinical and Experimental Immunology*.

[64] Crispin J. C., Oukka M., Bayliss G. (2008). Expanded double negative T cells in patients with systemic lupus erythematosus produce IL-17 and infiltrate the kidneys. *The Journal of Immunology*.

[65] Hedrich C. M., Crispin J. C., Rauen T. (2012). cAMP response element modulator alpha controls IL2 and IL17A expression during CD4 lineage commitment and subset distribution in lupus. *Proceedings of the National Academy of Sciences*.

[66] Fischer K., Voelkl S., Heymann J. (2005). Isolation and characterization of human antigen-specific TCR alpha beta+ CD4(-)CD8- double-negative regulatory T cells. *Blood*.

[67] Kattah N. H., Newell E. W., Jarrell J. A. (2015). Tetramers reveal IL-17-secreting CD4+ T cells that are specific for U1-70 in lupus and mixed connective tissue disease. *Proceedings of the National Academy of Sciences*.

[68] Dai H., He F., Tsokos G. C., Kyttaris V. C. (2017). IL-23 limits the production of IL-2 and promotes autoimmunity in lupus. *The Journal of Immunology*.

[69] Meng H., Zhao H., Cao X. (2019). Double-negative T cells remarkably promote neuroinflammation after ischemic stroke. *Proceedings of the National Academy of Sciences*.

[70] Alunno A., Bistoni O., Bartoloni E. (2013). IL-17-producing CD4-CD8- T cells are expanded in the peripheral blood, infiltrate salivary glands and are resistant to corticosteroids in patients with primary Sjogren’s syndrome. *Annals of the Rheumatic Diseases*.

[71] Sherlock J. P., Joyce-Shaikh B., Turner S. P. (2012). IL-23 induces spondyloarthropathy by acting on ROR-gammat+ CD3+CD4-CD8- entheseal resident T cells. *Nature Medicine*.

[72] Li H., Adamopoulos I. E., Moulton V. R. (2020). Systemic lupus erythematosus favors the generation of IL-17 producing double negative T cells. *Nature communications*.

[73] Chang Y., Al-Alwan L., Risse P. A. (2012). Th17-associated cytokines promote human airway smooth muscle cell proliferation. *The FASEB Journal*.

[74] Kyttaris V. C., Zhang Z., Kuchroo V. K., Oukka M., Tsokos G. C. (2010). Cutting edge: IL-23 receptor deficiency prevents the development of lupus nephritis in C57BL/6-lpr/lpr mice. *The Journal of Immunology*.

[75] Lee A. Y. S., Bannan J. L., Adams M. J., Korner H. (2017). Expression of CCR6 on B cells in systemic lupus erythematosus patients. *Clinical rheumatology*.

[76] Wen Z., Xu L., Xu W., Yin Z., Gao X., Xiong S. (2013). Interleukin-17 expression positively correlates with disease severity of lupus nephritis by increasing anti-double-stranded DNA antibody production in a lupus model induced by activated lymphocyte derived DNA. *PLoS One*.

[77] Hsu H. C., Yang P., Wang J. (2008). Interleukin 17-producing T helper cells and interleukin 17 orchestrate autoreactive germinal center development in autoimmune BXD2 mice. *Nature immunology*.

[78] Mitsdoerffer M., Lee Y., Jager A. (2010). Proinflammatory T helper type 17 cells are effective B-cell helpers. *Proceedings of the National Academy of Sciences*.

[79] Pisitkun P., Ha H. L., Wang H. (2012). Interleukin-17 cytokines are critical in development of fatal lupus glomerulonephritis. *Immunity*.

[80] Shivakumar S., Tsokos G. C., Datta S. K. (1989). T cell receptor alpha/beta expressing double-negative (CD4-/CD8-) and CD4+ T helper cells in humans augment the production of pathogenic anti-DNA autoantibodies associated with lupus nephritis. *The Journal of Immunology*.

[81] Frangou E., Chrysanthopoulou A., Mitsios A. (2019). REDD1/autophagy pathway promotes thromboinflammation and fibrosis in human systemic lupus erythematosus (SLE) through NETs decorated with tissue factor (TF) and interleukin-17A (IL-17A). *Annals of the Rheumatic Diseases*.

[82] Poissonnier A., Sanseau D., Le Gallo M. (2016). CD95-mediated calcium signaling promotes T helper 17 trafficking to inflamed organs in lupus-prone mice. *Immunity*.

[83] Turner J. E., Paust H. J., Steinmetz O. M. (2010). CCR6 recruits regulatory T cells and Th17 cells to the kidney in glomerulonephritis. *Journal of the American Society of Nephrology*.

[84] Wang J., Mizui M., Zeng L. F. (2016). Inhibition of SHP2 ameliorates the pathogenesis of systemic lupus erythematosus. *The Journal of clinical investigation*.

[85] Krebs C. F., Turner J. E., Paust H. J. (2016). Plasticity of Th17 cells in autoimmune kidney diseases. *The Journal of Immunology*.

[86] Maeda K., Kosugi T., Sato W. (2015). CD147/basigin limits lupus nephritis and Th17 cell differentiation in mice by inhibiting the interleukin-6/STAT-3 pathway. *Arthritis & Rheumatology*.

[87] Rozo C., Chinenov Y., Maharaj R. K. (2017). Targeting the RhoA-ROCK pathway to reverse T-cell dysfunction in SLE. *Annals of the rheumatic diseases*.

[88] Isgro J., Gupta S., Jacek E. (2013). Enhanced rho-associated protein kinase activation in patients with systemic lupus erythematosus. *Arthritis & Rheumatism*.

[89] Chatterjee M., Rauen T., Kis-Toth K. (2012). Increased expression of SLAM receptors SLAMF3 and SLAMF6 in systemic lupus erythematosus T lymphocytes promotes Th17 differentiation. *The Journal of Immunology*.

[90] Valdez P. A., Wang H., Seshasayee D. (2004). NTB-A, a new activating receptor in T cells that regulates autoimmune disease. *Journal of Biological Chemistry*.

[91] Karampetsou M. P., Comte D., Suarez-Fueyo A. (2019). Signaling lymphocytic activation molecule family member 1 engagement inhibits T cell-B cell interaction and diminishes interleukin-6 production and plasmablast differentiation in systemic lupus erythematosus. *Arthritis & Rheumatology*.

[92] Chatterjee M., Hedrich C. M., Rauen T., Ioannidis C., Terhorst C., Tsokos G. C. (2012). CD3-T cell receptor co-stimulation through SLAMF3 and SLAMF6 receptors enhances RORgammat recruitment to the IL17A promoter in human T lymphocytes. *Journal of Biological Chemistry*.

[93] Kyttaris V. C., Zhang Z., Kampagianni O., Tsokos G. C. (2011). Calcium signaling in systemic lupus erythematosus T cells: a treatment target. *Arthritis & Rheumatism*.

[94] Han L., Yang J., Wang X. (2014). The E3 deubiquitinase USP17 is a positive regulator of retinoic acid-related orphan nuclear receptor gammat (RORgammat) in Th17 cells. *Journal of Biological Chemistry*.

[95] Sato S., Zhang X. K., Temmoku J. (2020). Ets family transcription factor Fli-1 promotes leukocyte recruitment and production of IL-17A in the MRL/Lpr mouse model of lupus nephritis. *Cells*.

[96] Hofmann S. R., Mabert K., Kapplusch F. (2019). cAMP response element modulator alpha induces dual specificity protein phosphatase 4 to promote effector T cells in juvenile-onset lupus. *Journal of Biological Chemistry*.

[97] Voulgarelis M., Dafni U. G., Isenberg D. A., Moutsopoulos H. M. (1999). Malignant lymphoma in primary Sjogren's syndrome: a multicenter, retrospective, clinical study by the European Concerted Action on Sjogren’s Syndrome. *Arthritis & Rheumatism: Official Journal of the American College of Rheumatology*.

[98] Didier K., Bolko L., Giusti D. (2018). Autoantibodies associated with connective tissue diseases: what meaning for clinicians?. *Frontiers in immunology*.

[99] Theander E., Jonsson R., Sjostrom B., Brokstad K., Olsson P., Henriksson G. (2015). Prediction of Sjogren’s syndrome years before diagnosis and identification of patients with early onset and severe disease course by autoantibody profiling. *Arthritis & Rheumatology*.

[100] Espinosa A., Dardalhon V., Brauner S. (2009). Loss of the lupus autoantigen Ro52/Trim21 induces tissue inflammation and systemic autoimmunity by disregulating the IL-23-Th17 pathway. *Journal of experimental medicine*.

[101] Ysebrant de Lendonck L., Tonon S., Nguyen M. (2013). Interferon regulatory factor 3 controls interleukin-17 expression in CD8 T lymphocytes. *Proceedings of the National Academy of Sciences*.

[102] Yan J., Pandey S. P., Barnes B. J., Turner J. R., Abraham C. (2020). T cell-intrinsic IRF5 regulates T cell signaling, migration, and differentiation and promotes intestinal inflammation. *Cell reports*.

[103] Xue D., Shi H., Smith J. D. (2003). A lupus-like syndrome develops in mice lacking the Ro 60-kDa protein, a major lupus autoantigen. *Proceedings of the National Academy of Sciences*.

[104] Katsifis G. E., Rekka S., Moutsopoulos N. M., Pillemer S., Wahl S. M. (2009). Systemic and local interleukin-17 and linked cytokines associated with Sjogren’s syndrome immunopathogenesis. *The American journal of pathology*.

[105] Sun Y., Wang Y., Chen S. (2018). Expression of G*α*q Is Decreased in Lymphocytes from Primary Sjögren’s Syndrome Patients and Related to Increased IL-17A Expression. *Journal of Immunology Research*.

[106] Fei Y., Zhang W., Lin D. (2014). Clinical parameter and Th17 related to lymphocytes infiltrating degree of labial salivary gland in primary Sjogren’s syndrome. *Clinical rheumatology*.

[107] Fusconi M., Musy I., Valente D. (2020). Immunohistochemical detection of IL-17 and IL-23 improves the identification of patients with a possible diagnosis of Sjogren’s syndrome. *Pathology-Research and Practice*.

[108] Liu R., Gao C., Chen H., Li Y., Jin Y., Qi H. (2017). Analysis of Th17-associated cytokines and clinical correlations in patients with dry eye disease. *PLoS One*.

[109] Nguyen C. Q., Hu M. H., Li Y., Stewart C., Peck A. B. (2008). Salivary gland tissue expression of interleukin-23 and interleukin-17 in Sjogren’s syndrome: findings in humans and mice. *Arthritis & Rheumatism: Official Journal of the American College of Rheumatology*.

[110] Voigt A., Esfandiary L., Wanchoo A. (2016). Sexual dimorphic function of IL-17 in salivary gland dysfunction of the C57BL/6.NOD-Aec1Aec2 model of Sjogren’s syndrome. *Scientific reports*.

[111] Sakai A., Sugawara Y., Kuroishi T., Sasano T., Sugawara S. (2008). Identification of IL-18 and Th17 cells in salivary glands of patients with Sjogren’s syndrome, and amplification of IL-17-mediated secretion of inflammatory cytokines from salivary gland cells by IL-18. *The Journal of Immunology*.

[112] Nguyen C. Q., Yin H., Lee B. H., Carcamo W. C., Chiorini J. A., Peck A. B. (2010). Pathogenic effect of interleukin-17A in induction of Sjogren’s syndrome-like disease using adenovirus-mediated gene transfer. *Arthritis research & therapy*.

[113] Nguyen C. Q., Yin H., Lee B. H., Chiorini J. A., Peck A. B. (2011). IL17: potential therapeutic target in Sjogren’s syndrome using adenovirus-mediated gene transfer. *Laboratory investigation*.

[114] Lin X., Rui K., Deng J. (2015). Th17 cells play a critical role in the development of experimental Sjogren’s syndrome. *Annals of the rheumatic diseases*.

[115] Uddin M., Polley-Mandal M., Beg O. U. (2003). Kallikrein-like prorenin-converting enzymes in inbred hypertensive mice. *Biochemical and biophysical research communications*.

[116] Takada K., Takiguchi M., Konno A., Inaba M. (2005). Autoimmunity against a tissue kallikrein in IQI/Jic mice: a model for Sjogren’s syndrome. *Journal of Biological Chemistry*.

[117] Moustardas P., Yamada-Fowler N., Apostolou E., Tzioufas A. G., Turkina M. V., Spyrou G. (2021). Deregulation of the kallikrein protease family in the salivary glands of the Sjogren’s syndrome ERdj5 knockout mouse model. *Frontiers in immunology*.

[118] Wu C., Wang Z., Zourelias L., Thakker H., Passineau M. J. (2015). IL-17 sequestration via salivary gland gene therapy in a mouse model of Sjogren’s syndrome suppresses disease-associated expression of the putative autoantigen Klk1b22. *Arthritis research & therapy*.

[119] Zhang L. W., Cong X., Zhang Y. (2016). Interleukin-17 impairs salivary tight junction integrity in Sjogren’s syndrome. *Journal of dental research*.

[120] Ciccia F., Guggino G., Rizzo A. (2014). Rituximab modulates IL-17 expression in the salivary glands of patients with primary Sjogren’s syndrome. *Rheumatology (Oxford).*.

[121] Lucchesi D., Coleby R., Pontarini E. (2020). Impaired Interleukin‐27–Mediated Control of CD 4+ T Cell Function Impact on Ectopic Lymphoid Structure Formation in Patients With Sjögren's Syndrome. *Arthritis & Rheumatology*.

[122] Weng X., Liu Y., Cui S., Cheng B. (2018). The role of RORalpha in salivary gland lesions in patients with primary Sjogren’s syndrome. *Arthritis Research & Therapy*.

[123] Park E., Kim D., Lee S. M., Jun H. S. (2017). Inhibition of lysophosphatidic acid receptor ameliorates Sjogren’s syndrome in NOD mice. *Oncotarget*.

[124] Denton C. P., Khanna D. (2017). Systemic sclerosis. *Lancet*.

[125] Okamoto Y., Hasegawa M., Matsushita T. (2012). Potential roles of interleukin-17A in the development of skin fibrosis in mice. *Arthritis & Rheumatism*.

[126] Yang X., Yang J., Xing X., Wan L., Li M. (2014). Increased frequency of Th17 cells in systemic sclerosis is related to disease activity and collagen overproduction. *Arthritis research & therapy*.

[127] Cascio S., Medsger T. A., Hawse W. F. (2018). 14-3-3z sequesters cytosolic T-bet, upregulating IL-13 levels in TC2 and CD8(+) lymphocytes from patients with scleroderma. *Journal of Allergy and Clinical Immunology*.

[128] Goncalves R. S. G., Pereira M. C., Dantas A. T. (2018). IL-17 and related cytokines involved in systemic sclerosis: perspectives. *Autoimmunity*.

[129] Xu W., Su L., Qing P. (2017). Elevated levels of TL1A are associated with disease activity in patients with systemic sclerosis. *Clinical Rheumatology*.

[130] Xing X., Yang J., Yang X. (2013). IL-17A induces endothelial inflammation in systemic sclerosis via the ERK signaling pathway. *PLoS One*.

[131] Murata M., Fujimoto M., Matsushita T. (2008). Clinical association of serum interleukin-17 levels in systemic sclerosis: is systemic sclerosis a Th17 disease?. *Journal of dermatological science*.

[132] Nakashima T., Jinnin M., Yamane K. (2012). Impaired IL-17 signaling pathway contributes to the increased collagen expression in scleroderma fibroblasts. *The Journal of Immunology*.

[133] Komura K., Fujimoto M., Hasegawa M. (2008). Increased serum interleukin 23 in patients with systemic sclerosis. *The Journal of Rheumatology*.

[134] Truchetet M. E., Brembilla N. C., Montanari E. (2013). Interleukin-17A+ cell counts are increased in systemic sclerosis skin and their number is inversely correlated with the extent of skin involvement. *Arthritis & Rheumatism*.

[135] Kurasawa K., Hirose K., Sano H. (2000). Increased interleukin-17 production in patients with systemic sclerosis. *Arthritis & Rheumatism*.

[136] Koumakis E., Bouaziz M., Dieude P. (2013). A regulatory variant in CCR6 is associated with susceptibility to antitopoisomerase-positive systemic sclerosis. *Arthritis & Rheumatism*.

[137] Brembilla N. C., Montanari E., Truchetet M. E., Raschi E., Meroni P., Chizzolini C. (2013). Th17 cells favor inflammatory responses while inhibiting type I collagen deposition by dermal fibroblasts: differential effects in healthy and systemic sclerosis fibroblasts. *Arthritis research & therapy*.

[138] Dufour A. M., Alvarez M., Russo B., Chizzolini C. (2018). Interleukin-6 and type-I collagen production by systemic sclerosis fibroblasts are differentially regulated by interleukin-17A in the presence of transforming growth factor-beta 1. *Frontiers in Immunology*.

[139] Dufour A. M., Borowczyk-Michalowska J., Alvarez M. (2020). IL-17A dissociates inflammation from fibrogenesis in systemic sclerosis. *Journal of Investigative Dermatology*.

[140] Liu M., Yang J., Xing X., Cui X., Li M. (2014). Interleukin-17A promotes functional activation of systemic sclerosis patient-derived dermal vascular smooth muscle cells by extracellular-regulated protein kinases signalling pathway. *Arthritis research & therapy*.

[141] Akhmetshina A., Beer J., Zwerina K. (2010). Decreased lymphatic vessel counts in patients with systemic sclerosis: association with fingertip ulcers. *Arthritis research & therapy*.

[142] Bisoendial R., Tabet F., Tak P. P. (2015). Apolipoprotein A-I limits the negative effect of tumor necrosis factor on lymphangiogenesis. *Arteriosclerosis, Thrombosis, and Vascular Biology*.

[143] Park H. J., Yuk C. M., Shin K., Lee S. H. (2018). Interleukin-17A negatively regulates lymphangiogenesis in T helper 17 cell-mediated inflammation. *Mucosal Immunology*.

[144] Sumida T., Sakamoto A., Murata H. (1995). Selective reduction of T cells bearing invariant V alpha 24J alpha Q antigen receptor in patients with systemic sclerosis. *The Journal of experimental medicine*.

[145] Sakamoto A., Sumida T., Maeda T. (1992). T cell receptor V beta repertoire of double-negative alpha/beta T cells in patients with systemic sclerosis. *Arthritis & Rheumatism: Official Journal of the American College of Rheumatology*.

[146] Tiev K. P., Abriol J., Burland M. C. (2005). T cell repertoire in patients with stable scleroderma. *Clinical & Experimental Immunology*.

[147] De Palma R., Del Galdo F., Lupoli S., Altucci P., Abbate G., Valentini G. (2006). Peripheral T lymphocytes from patients with early systemic sclerosis co-cultured with autologous fibroblasts undergo an oligoclonal expansion similar to that occurring in the skin. *Clinical & Experimental Immunology*.

[148] Liu X., Gao N., Li M. (2013). Elevated levels of CD4(+)CD25(+)FoxP3(+) T cells in systemic sclerosis patients contribute to the secretion of IL-17 and immunosuppression dysfunction. *PLoS One*.

[149] Hasegawa M., Fujimoto M., Matsushita T., Hamaguchi Y., Takehara K. (2013). Augmented ICOS expression in patients with early diffuse cutaneous systemic sclerosis. *Rheumatology (Oxford)*.

